# Tracking CTC Dynamics: Trop2 and EpCAM in Metastatic Breast Cancer Progression

**DOI:** 10.3390/cancers17233885

**Published:** 2025-12-04

**Authors:** Álvaro González, Estibaliz Alegre

**Affiliations:** 1 Service of Biochemistry, Cancer Center Clínica Universidad de Navarra (CCUN), Av. Pío XII 36, 31008 Pamplona, Spain; ealegre@unav.es; 2 IdiSNA—Navarra Institute for Health Research, 31008 Pamplona, Spain

Breast cancer is the most prevalent malignancy worldwide and a leading cause of cancer-related mortality, primarily due to distant metastasis [1]. The three subtypes—hormone receptor-positive (HR+), HER2-positive (HER2+), and triple-negative breast cancer (TNBC)—differ markedly in prognosis and metastatic behavior. HR+ disease generally has the best prognosis. In contrast, TNBC is the most aggressive subtype, with high invasiveness, propensity for distant metastasis, and limited treatment options [2]. HER2+ disease presents an intermediate metastatic risk, and its outcomes have improved with targeted therapies [3].

Circulating tumor cells (CTCs) are key mediators of distant metastasis and be used as minimally invasive biomarkers for disease monitoring. However, their rarity and heterogeneity across subtypes, epithelial-to-mesenchymal transition (EMT) status, and primary tumor versus metastatic origin complicate the interpretation of analytical results [4]. Classical CTCs are defined as positive for the epithelial markers EpCAM and cytokeratins (CKs), and negative for the leukocyte marker CD45. Both EpCAM and its homolog Trop2 facilitate transient cell–cell adhesion [5] and Merkley et al. [6] have analyzed them as CTC biomarkers. Classic CTCs (EpCAM+CK+) or Trop2+CK+ CTCs (T2CTCs) occur as single cells or clusters and are sometimes associated with CD45+ immune cells. Cluster formation significantly enhances metastatic competency by conferring resistance to shear stress, immune attack, and anoikis.

CTC detection methods have evolved beyond mere enumeration. CellSearch^®^, the only FDA-cleared platform, relies on EpCAM-based immunomagnetic capture, which misses EpCAM-low or EMT-like CTCs [7]. However, newer technologies, such as RareCyte^®^, Parsortix, and microfluidic systems, integrate size-based enrichment and multi-marker staining. This improves sensitivity for heterogeneous populations and enables visualization of clusters. These advances are relevant for the accurate characterization of CTC biology and its clinical utility.

Merkley et al. [6], using RareCyte, applied longitudinal linear mixed modeling to 272 samples from 51 metastatic breast cancer patients, showing that EpCAM and Trop2 are central to clustering. EpCAM strongly correlates with heterotypic clusters containing CD45^+^ cells, while Trop2 correlates with homotypic clustering. Cluster size and CD45+ association were associated with metastatic competency.

Longitudinal analysis revealed distinct biomarker trajectories following a metastatic diagnosis [8]. In HER2+ cancers, EpCAM+ CTCs and clusters of more than two cells increased over time, while they remained stable in HR+ patients [6]. On the other hand, Trop2+ CTCs showed a baseline effect in bone metastasis, and larger clusters were associated with brain metastasis in HER2+ patients. These findings underscore the clinical relevance of monitoring CTC dynamics. For example, in HER2+ patients, monitoring CTC dynamics may enable early CNS-active treatment interventions to prevent brain metastasis.

Merkley et al. acknowledge the limitations of the small size of the Trop2 datasets and the marginal significance of some analyses. The role of CK19 in clustering remains unclear, and other adhesion-related biomarkers, such as placoglobin, CD44, and ICAM, were not evaluated. Additionally, these findings emphasize the need for biomarker-driven strategies that integrate EpCAM/Trop2 profiling with CNS-active HER2-targeted therapies, aiming to intercept micrometastatic disease before clinical progression.

Future research should expand biomarker panels [9], explore EpCAM/Trop2 mechanisms in organotropism, and integrate multi-omic profiling with standardized protocols to enable early therapeutic strategies targeting clusters. Longitudinal CTC dynamics [6,7,8] could transform the management of metastatic breast cancer by enabling early, precise interventions to prevent the metastatic cascade.
